# Discovery of tumor antigens in renal cell carcinoma and prospects for clinical application

**DOI:** 10.3389/fimmu.2026.1771006

**Published:** 2026-03-27

**Authors:** Evelina Kagirova, Kadriia Enikeeva, Radmir Mukhamadeev, Dilara Asadullina, Yuliya Sharifyanova, Polina Shmelkova, Diana Gainullina, Daria Trifonova, Pia Gattinger, Guillermo Docena, Alexandra Dubovets, Inna Tulaeva, Alexander Karaulov, Rudolf Valenta, Valentin Pavlov

**Affiliations:** 1 Institute of Urology and Clinical Oncology, Bashkir State Medical University, Ufa, Russia; 2 Division of Immunopathology, Department of Pathophysiology and Allergy Research, Medical University of Vienna, Vienna, Austria; 3 Laboratory of Immunopathology, Department of Clinical Immunology and Allergology, Sechenov First Moscow State Medical University, Moscow, Russia; 4 Laboratory of Immunology and Infectiology, Life Improvement by Future Technologies (LIFT) Center, Moscow, Russia; 5 Instituto de Estudios Inmunológicos y Fisiopatológicos-Instituto de Estudios Inmunológicos y Fisiopatológicos (IIFP), Facultad de Ciencias Exactas, Universidad Nacional de La Plata, Consejo Nacional de Investigaciones Científicas y Técnicas (CONICET), La Plata, Argentina; 6 Center for Molecular Allergology, Karl Landsteiner University for Healthcare Sciences, Krems, Austria

**Keywords:** antigens, cancer, immunotherapies, renal cell carcinoma, treatment, tumor, vaccine

## Abstract

Renal cell carcinoma (RCC) is a prevalent and heterogeneous malignancy with clear cell RCC (ccRCC) as the most common subtype. Despite advances in surgical, targeted, and immunotherapeutic approaches, prognosis for advanced and metastatic RCC remains poor, and the effectiveness of current immunotherapies is limited by immune tolerance, tumor heterogeneity, and adverse effects. The identification of tumor-associated antigens (TAAs) and tumor-specific antigens (TSAs) is crucial for the development of personalized and effective antigen-directed therapies, including vaccines, antibodies and adoptive cell therapies. This review summarizes epidemiological data, molecular features of RCC, and the role of the von Hippel Lindau – Hypoxia Inducible Factor (VHL-HIF) signaling pathway in pathogenesis, alongside recent progress in characterizing possible antigen targets for vaccination such as TOP2A, NCF4, FMNL1, DOK3, MUC1, CAIX, CD70, and 5T4. Preclinical models, including genetically engineered mouse models, zebrafish, and various patient-derived xenograft (PDX) systems, are discussed as tools for studying tumor biology and testing immunotherapeutic strategies. Clinical trial data on RCC vaccines, including autologous renal tumor cell vaccination, peptide-based, dendritic cell-based, and viral vector platforms, demonstrate immunogenicity but have not yet yielded clear survival benefits in phase III trials. Future directions emphasize integrating antigen discovery with immune profiling, refining preclinical modeling, and developing personalized vaccines to enhance therapeutic efficacy, particularly for immunologically favorable patient subtypes and for certain applications such as reducing metastasis after surgery. In particular, we discuss carrier-based vaccine approaches for overcoming tolerance and increasing the immunogenicity of vaccines.

## 1 Introduction

Renal cell carcinoma (RCC) represents the 15th most common cancer as shown by the GLOBOCAN statistics (1, 2). Some epidemiological facts: In 2020, there were 431,000 new cases of kidney cancer worldwide (271,000 in males and 160,000 in females). In 2020, the global age-standardized incidence rate (ASIR) of kidney cancer was 4.6/100,000, of which 6.1/100,000 were males and 3.2/100,000 were females. The ASIR of kidney cancer in males in different human development index (HDI) countries is higher than that in females. The ASIR of kidney cancer in countries with extremely high and high HDI is higher than that in countries with medium and low HDI (3).

Among RCC, the clear cell RCC (ccRCC) has an incidence of 75%, followed by papillary RCC (pRCC, 15-20% of cases), chromophobe RCC (chRCC, 5% of cases) and other more rare subtypes (4, 5). Risk factors for ccRCC include genetic factors such as Von Hippel-Lindau (VHL) disease and tuberous sclerosis complex (TSC) as well as modifiable factors such as smoking, obesity and hypertension (6).

RCC is strongly associated with structural alterations in the 3p21 chromosome (short arm), particularly with 3p loss, where the VHL tumor suppressor gene is located, however, the 3p loss is not present in all RCC subtypes. The Cancer Genome Atlas (TCGA) research network showed a remodeling of cellular metabolism in ccRCC involving dysregulation of tricarboxylic acid cycle and phosphate pathway genes, and decreased 5′-Adenosine monophosphate-activated protein kinase (AMPK), which indicates a poor prognosis (7). A plethora of gene variants are related to RCC including the tumor suppressor VHL gene whose epigenetic alterations are present in 8 out of 10 of ccRCC cases. The VHL gene is essential for cellular oxygen detection by targeting Hypoxia-Inducible Factors (HIFs) for ubiquitination and subsequent degradation. When VHL mutations occur, its Tumor-suppressing function is lost and HIF is over-expressed, leading to the over-expression of vascular endothelial growth factor (VEGF) and platelet-derived growth factor (PDGF) among other genes. Therefore, these events result in uncontrolled/abnormal angiogenesis, increased tumor growth and invasion (8).

Sequencing studies revealed additional driver genes that are involved in the development of RCC such as the protein polybromo-1 (PBRM1), ubiquitin carboxyl-terminal hydrolase BAP1, histone Methyltransferase SETD2, protein elongin-c known as TCEB1, and the enzyme lysine-specific demethylase encoded by the KDM5C gene (9). The consequences of these mutations remain largely unknown at the clinical level due to the scarcity of samples, the inadequate follow up of the patients and the lack of diagnostic markers. The observed mutations include VHL, PBRM1, BAP1, and SETD2 as well as histone modifiers (Lysine-specific demethylase 5a: KDM5a; AT-rich interactive domain-containing protein 1a: ARID1a, and Lysine-specific demethylase 6A: UTX). Particularly, SETD2 mutations are correlated with alterations in chromatin packaging. Moreover, mutations in genes related to the mTOR pathway (PIK3CA, PTEN, and mTOR) are also observed in tumor samples (9, 10).

Early diagnosis of ccRCC is a major challenge and current treatment options include radical/partial nephrectomy, tyrosine kinase inhibitors and/or immune checkpoint inhibitors (11, 12) and mammalian target of rapamycin (mTOR) inhibitors. Chemotherapy shows low efficacy in ccRCC, which often forms local metastases and spreads hematogenously by invasion of blood vessels (6).

Treatment of kidney malignancies varies and is influenced by the tumor size and the presence of metastatic sites. Patient life expectancy of the patients is 5 to 10 years or shorter if metastatic sites are present. In brief, the main treatments of kidney cancer include: a) full or partial nephrectomy, b) cryotherapy or radiofrequency ablation in which cancer cells are destroyed via freezing or heating, c) radiotherapy using high-energy ionizing radiation to destroy cancer cells, d) embolization which represents a minimally invasive procedure that blocks one or more blood vessels limiting tumor blood supply, e) anti-angiogenic agents (sunitinib, sorafenib and temsirolimus), and f) immunotherapy (7).

Immunotherapy is currently one of the most promising areas of research in cancer therapeutics. Immunity has a pivotal role in cancer progression favoring either destruction or stimulation of cancer cells in association with the tumor microenvironment, a process called cancer “immunoediting” (8). Most patients still do not receive long-term benefit from these therapies, and many experience immune-related side effects such as immune-mediated nephritis, pneumonitis, hepatitis, hypophysitis, thyroiditis, and cytokine storm (13, 14).

To improve the efficacy and safety of renal cancer immunotherapy, it is important to identify antigens that can be specifically targeted by immunotherapy. The study of tumor-specific antigens and tumor-associated antigens is therefore relevant for the development of tumor vaccines. Accordingly, it is suggested that the identification of antigens in RCC will improve the understanding of response and resistance to current therapies and will be the basis for the development of antigen-directed immunotherapy strategies (15).

## 2 Tumor-specific antigens and tumor-associated antigens: definition and characteristics

The initial and crucial step in developing cancer vaccines is selecting an antigen that exhibits high tumor specificity and is capable of eliciting strong and manageable antitumor humoral and cellular immune responses (16). Tumor antigens are categorized into tumor-associated antigens (TAAs) and tumor-specific antigens (TSAs) based on their tissue distribution, expression levels, and central tolerance status (17–20).

TAAs are typically overexpressed in tumors, while also being present in normal tissues. They show limited tumor specificity, high central tolerance, and low immunogenicity, encompassing mainly tissue differentiation antigens and carcinoembryonic antigens (21). The central immune tolerance of TAAs poses a significant obstacle in designing cancer vaccines targeting these antigens. Due to this problem the clinical trend is shifting towards using combinations of multiple shared TAAs in the development of targeted cancer vaccines. Such TAAs are broadly expressed in related tumors and can trigger antitumor immune responses when paired with various vectors or adjuvants (22).

TSAs are typically neoantigens generated by nonsynonymous mutations in the genome of tumor cells, which gives them high tumor specificity, strong immunogenicity, and there is weak central tolerance (21, 23). Several studies have confirmed a link between TSAs and antitumor immune responses. An analysis of thousands of RNA sequences from 18 different solid tumors in TCGA revealed a positive correlation between the number of neoantigens in each tumor and the expression of genes related to T cell cytotoxic activity (24). Similarly, an analysis of RNA-seq data from six tumor types in 515 patients showed that a higher level of immunogenic mutant epitopes was associated with better patient survival (25). Tumors with high levels of immunogenic mutations have been observed to exhibit significantly elevated expression of T-cell surface glycoprotein CD8 alpha chain (CD8A), programmed cell death 1 protein (PD-1), and cytotoxic T-lymphocyte-associated Protein 4 (CTLA4), which may potentially render them more sensitive to checkpoint inhibitor therapies such as anti-CTLA-4 and anti-PD1. However, these findings are not universally confirmed, and the increased responsiveness to checkpoint blockade remains to be unequivocally established (25).

Moreover, tumors rich in neoantigens were found to be more homogenous compared to those with fewer neoantigens (26). Tumors with a mutational load of more than 10 somatic mutations per million bases (equivalent to 150 nonsynonymous mutations in expressed genes) are more likely to produce immunogenic neoantigens, while tumors with a mutational load below 1 somatic mutation per million bases are less likely to do so. Most tumors exhibit a mutational load between 1 and 10 somatic mutations per million bases, making them generally capable of producing neoantigens that can be recognized by T cells (27). Rajasagi et al. further analyzed predicted mutant human leukocyte antigen (HLA)-binding peptides from 13 different tumor types (2,488 samples) using whole-exome sequencing and the NetMHCpan HLA-peptide binding prediction algorithm, demonstrating that each tumor could generate tens to thousands of neoantigens, suggesting that neoantigens are common across most tumors (28).

Four key criteria are relevant for considering a TAA as a possible target for a T cell-based vaccine.

1. Absence of pre-existing immune tolerance: A functional immune system is essential for a vaccine’s efficacy. Vaccines targeting TAAs that may be self-antigens (e.g., HER2 in breast cancer) could potentially break immune tolerance to these self-proteins. However, given the genetic instability of cancers, it may be more feasible to target TAAs that have become neo-antigens through mutations, making them unrecognized as “self” by the immune system (29).
2. Immunogenicity: TAAs should be capable of inducing a specific memory CD8+ T-cell response and antibody responses. Such responses can be measured through assays like ELISPOT, carboxyfluorescein-diacetate-succinimidylester (CFSE) staining (30) or FluoroSpot to detect TAA-specific T cells, and assays measuring specific antibody responses, respectively.
3. Differential expression between tumor and normal tissues: Toxicity from immunotherapy and spontaneous autoimmune paraneoplastic syndromes are believed to result from TAA expression in normal tissues. Therefore, the uncertain expression patterns of antigens in normal tissues highlight the need for thorough monitoring of potential toxicity.
4. Role in tumorigenesis and tumor cell survival: Because tumors are genomically diverse and inherently unstable, an ideal TAA should be involved in an oncogenic addictive pathway, reducing the risk of selecting a poorly immunogenic variant during the vaccine-induced immune response. For example, in a phase I trial of a vaccine targeting a VHL-mutated antigen, T cells from 4 out of 5 patients showed reactivity against the mutated peptides using IFN-γ ELISPOT assays. Unfortunately, the phase II study (NCT00001703) yielded inconclusive results due to insufficient patient enrollment (29).
5. Surface expression on tumor cells: Regarding tumor vaccines focusing on the induction of tumor-specific antibodies capable of eliciting antibody-dependent cytokine release (ADCR), antibody-dependent cellular cytotoxicity (ADCC), antibody-dependent cellular phagocytosis (ADCP) and antibody-dependent complement activation (ADCA), it will be useful if the tumor antigen is expressed specifically on the surface of tumor cells (31).

### 2.1 Identification of TSA and TAA

To identify potential tumor antigens for ccRCC, a differential gene expression analysis between normal and malignant renal parenchymal tissues was conducted, resulting in the identification of 1,638 overexpressed genes with the potential to encode tumor-associated antigens. Further analysis of variations in tumor mutation counts and altered genome fractions in ccRCC cases was performed to screen for mutated TAA-encoding genes, yielding a total of 11,686 mutated genes. Among these, mutational analysis highlighted the top 10 genes with the highest mutation counts: Growth Arrest-Specific 8 antisense RNA 1 (GAS8-AS1), VHL tumor suppressor, mucin 16 (MUC16), ankyrin repeat and PH domain 3 (ARAP3), heme binding protein 1 (HEBP1), phosphatase and actin regulator 1 (PHACTR1), SSX family member 3 (SSX3), teashirt zinc finger homeobox 3 (TSHZ3), ATP binding cassette subfamily A member 6 (ABCA6), and CD4 molecule (CD4). The top 10 genes with the highest levels of genomic alterations included VHL tumor suppressor, inhibitor of nuclear factor kappa B kinase subunit beta (IKBKB), transforming acidic coiled-coil containing protein 1 (TACC1), neuregulin 1 (NRG1), protein O-mannose kinase (POMK), pleckstrin and Sec7 domain containing 3 (PSD3), polybromo 1 (PBRM1), adrenoceptor beta 3 (ADRB3), adaptor-related protein complex 3 subunit mu 2 (AP3M2), and cholinergic receptor nicotinic beta 3 subunit (CHRNB3). Altogether, 629 overexpressed and mutated genes were identified (32).

By analyzing expression, mutation, survival, and correlation data from The Cancer Genome Atlas Kidney Renal Clear Cell Carcinoma (TCGA-KIRC) dataset (https://www.cancerimagingarchive.net/collection/tcga-kirc/), four genes—DNA topoisomerase II alpha (TOP2A), neutrophil cytosol factor 4 (NCF4), formin-like protein 1 (FMNL1), and docking protein 3 (DOK3)—were identified as potential KIRC-specific neoantigen candidates. These genes were found to be upregulated, mutated, and positively correlated with both patient survival and antigen-presenting cells in TCGA-KIRC. Additionally, two immune subtypes, named RCC immune subtype (RIS) 1 and RIS2, were identified in KIRC. Patients classified under RIS2 showed better survival outcomes compared to those in RIS1. Further immune profiling revealed that RIS1 is an immunologically “hot” but immunosuppressive subtype, while RIS2 represents an immunologically “cold” phenotype. RIS1 and RIS2 also differed significantly in terms of tumor-infiltrating immune cells and immune checkpoint-related genes. Moreover, constructing the immune landscape for each KIRC patient helped to determine the composition of immune cell populations, predict survival outcomes, and guide the development of personalized mRNA vaccines. In conclusion, this study identified TOP2A, NCF4, FMNL1, and DOK3 as promising neoantigen targets for KIRC vaccine development, with RIS2 patients potentially benefiting more from vaccination strategies (33).

At the same time, the level of scientific development does not yet allow to achieve a positive therapeutic effect of vaccination in all or most of the patients. The success rate in most clinical trials is quite low. There are no reliable criteria to accurately predict which kidney cancer patients will benefit from TAA/TSA vaccination. For this reason, more research is needed to identify TSA and TAA and to investigate the efficacy of TAA/TSA-based vaccination therapy in kidney cancer.

### 2.2 Natural anti-tumoral immune response against tumor antigens

The immune system can continuously recognize and eliminate tumor cells through several coordinated mechanisms involving both the innate (NK cells, M1 macrophages, dendritic cells, and the complement system) and adaptive (CD8+ cytotoxic T cells, CD4+ T cells, B cells and antibodies, and memory B and T cells) arms of immunity. It continuously monitors tissues to identify and remove transformed cells before they become clinically detectable, a protective process known as immunological surveillance. Failures in this surveillance can allow tumors to develop and progress.

The concept of immunological surveillance was first formally proposed by Frank Macfarlane Burnet around 1960, when he described the clonal selection theory and the immune system’s role in identifying and eliminating emerging malignant cells. It was later recognized that tumor cells often express tumor-specific antigens and tumor-associated antigens, which the immune system can identify as abnormal. Today, immunological surveillance is considered to be a part of the broader concept of cancer immunoediting, which includes the “Three E’s” phases: Elimination (the classic surveillance phase), Equilibrium (immunological control of residual cells), and Escape (tumor cells develop mechanisms to avoid immune detection). This concept was coined by Robert Schreiber, together with Lloyd Old and Mark Smyth, in 2002 (34). Tumor vaccines aim to stimulate the immune system to recognize and attack cancer cells by presenting tumor antigens in a way that elicits a strong immune response as described.

## 3 *In vivo* models for ccRCC

Genetically engineered mouse (GEM) models can replicate the initiation, progression, and metastasis of cancer, making them valuable for understanding tumor development and serving as ideal preclinical platforms for drug testing and biomarker identification (35).

Genetically engineered animal models have proven to be powerful tools for studying tumorigenesis and serve as preclinical models for drug testing, which is often challenging or unfeasible to conduct clinically (36). While GEM models have been successfully created for various cancers (37), attempts to develop autochthonous models of ccRCC that accurately replicate its key molecular and cellular characteristics have faced significant challenges, despite the fact that ccRCC is one of the few human cancers known to arise from a specific gene mutation (38, 39). However, recent years have seen remarkable progress in overcoming these obstacles (35).

Proximal tubular epithelial cells, widely regarded as the cellular origin of ccRCC, have been the primary choice for conditional targeting in kidney studies. Rankin and colleagues created the first conditional knockout mouse model by using the phosphoenolpyruvate carboxykinase-Cre system to inactivate the VHL gene specifically in renal proximal tubule cells. This model exhibited cellular proliferation, lipid accumulation, and, notably, macroscopic renal cyst formation at a rate of 18% in mice older than one year. However, no RCC development was observed until the mice reached 25 months of age. Although VHL-related renal cysts are generally viewed as preneoplastic lesions, this model does not fully represent a model suitable for RCC research (**Table 1**) (40).

**Table 1:** *In vivo* models for research in RCC.

*In vivo* model	Description	Advantages	Deficiencies	Ref.
VHL-knockout mice	Application of the phosphoenolpyruvate carboxykinase-Cre system to inactivate the VHL gene specifically in renal proximal tubule cells.	In this model, cell proliferation, lipid accumulation and, importantly, macroscopic renal cyst formation were observed at a frequency of 18% in mice older than one year of age. The cysts formed share morphologic and molecular features with VHL-associated renal cysts.	The development of RCC was not observed until the mice reached 25 months of age.	(40)
	Mouse model using Hoxb7-Cre to delete VHL in collecting duct cells and parts of the distal tubules. Hoxb7–Cre–EGFP is an independently generated line expressing Cre and EGFP on the same bicistronic message driven by the Hoxb7 promoter. Inactivation of VHL in cells of the collecting ducts and part of the distal tubules.	The lesions are cystic, characterized by pronounced fibrosis and significant hyperplasia. A large number of infiltrating macrophages and lymphocytes are detected.	No further progression of the cancer	(41)
	Used the Pax8-Cre system to inactivate VHL throughout the tubule system. VHL knockout (VHL2/2) model, which was implied by the Pax8 promoter and confers an inducible Cre-driven knockout in the complete tubular system of the kidney	Tubular expression of HIF2a (and HIF-1a) in the tubules	There was no indication of RСС	(42)
	Transgenic mice with conditional knockout of VHL in TALs using a Cre-recombinase driven by the Tamm-Horsfall protein (Thp) promoter, which is selectively expressed in TALs. THP-Cre system, targets the medullary thick ascending loop of Henle and early distal tubule.	These mice showed strong expression of HIF-1 in TALs (thick ascending limb)	No changes in kidney morphology or function under control conditions.	(43)
VHL knockout zebrafish	Vhlhu2117 fish	The VHL -/- zebrafish kidney is characterized by increased tubule diameter, disorganized cilia, dramatic formation of cytoplasmic lipid vesicles, glycogen accumulation, aberrant cell proliferation, and abnormal apoptosis.	VHL loss alone is not sufficient to initiate kidney tumor formation	(44)
PDX	Patient-derived tumor models (PDTM).	Tumor grafts retain the histology, gene expression, DNA copy number changes, and more than 90% of the protein-coding gene mutations of the corresponding tumors.	These models do not take into account the complexities of tumor-immune cell interactions, and therefore these critical parameters are often missing or underrepresented in studies with these models.	(45)
	Orthotopic xenografted RCC tumor of the humanized mouse NOD/SCID/IL2Rγ followed by additional injection of allogeneic human peripheral blood mononuclear cells	This model is capable of mediating the human immune response *in vivo*, including tumor infiltration by NK cells and T-cell activation, resulting in inhibition of CAIX tumor growth. The orthotopic xenografted humanized mouse tumor represents an improved model to evaluate the *in vivo* antitumor potential of a fully human monoclonal antibody (mAb) for RCC therapy.	No human NK cells are preserved in the model. But otherwise the interaction with immune cells is better revealed in this model than in the patient-derived tumor models (PDTM)	(46)
	HU-PDX. Humanized patient-derived xenograft (PDX) model using autologous CD34+ cells from bone marrow aspirate obtained from a patient with metastatic clear cell renal cell carcinoma (mRCC) from whom a PDX had been developed.	HPSCs derived from a single bone marrow aspirate can restore the immune system in mice, enabling agents like nivolumab to suppress tumor growth in PDX models and induce durable remission. This process leads to the recovery of human T cells, B cells, and NK cells; moreover, unlike humanized mouse models generated with umbilical cord blood, this system avoids tumor rejection caused by HLA mismatch. Importantly, in studies where both the tumor and the bone marrow cells originate from the same patient, the model more accurately reflects anti-tumor immunity rather than transplantation rejection, thereby increasing its translational relevance for immunotherapy research.		(47)

Noonan and colleagues also sought to create a zebrafish larvae model alongside their mouse model. The proximal pronephric tubule of the VHL−/− zebrafish kidney exhibited distinct clear cell histology, including tubule dilation, disorganized cilia, glycogen buildup, and abnormal cell proliferation, making it a potential model for studying early-stage RCC. Notably, their zebrafish model demonstrated promising outcomes with the novel hypoxia-inducible factor 2 alpha (HIF2a) inhibitor treatment (**Table 1**) (44).

Frew et al. used the Ksp1.3-Cre system (which also affects proximal tubules) to create a conditional knockout of VHL in the distal tubules and collecting ducts, but observed no abnormalities except for hydronephrosis (48). More recently, Pritchett and colleagues developed a mouse model using Hoxb7-Cre to delete VHL in collecting duct cells and parts of the distal tubules. This model showed significant tubular abnormalities, such as dilation, hyperplasia, presence of clear cells, cysts, disrupted structure, interstitial fibrosis, and inflammation, yet no ccRCC development was observed (**Table 1**) (41). Similarly, other researchers employed the Pax8-Cre system to inactivate VHL across the entire tubular system (**Table 1**) (42) or the THP-Cre system targeting the medullary thick ascending loop of Henle and early distal tubule, but found no evidence of RCC or renal cyst formation (**Table 1**) (43). These findings suggest that VHL loss alone is not sufficient to initiate kidney tumor formation (35).

The recently developed GEM models of ccRCC may offer promise for investigating biological mechanisms of tumor progression, discovering new biomarkers, and testing novel therapies – in areas currently dominated by *in vitro* studies and murine xenograft models based on RCC cell lines or human ccRCC explants. These GEM models are eventually useful for research on emerging RCC immunotherapies, which cannot be effectively studied using *in vitro* cell lines or immune-compromised xenograft mouse models. An autochthonous mouse model provides opportunities to evaluate therapeutic efficacy and identify relevant biomarkers within a genetically controlled environment, preserving an intact tumor microenvironment and immune response (35).

The patient-derived xenograft (PDX) model is not a recent development, but it has consistently shown a strong correlation between responses in the model system and those observed in clinical settings (49). The first successful heterotransplantation of a human tumor into mice was achieved in 1969 using colon cancer and athymic nude mice (50). This model offers numerous advantages, such as maintaining tumor histology, fast implementation, and simplicity of use for researchers. The fundamental premise of the PDX model is that it preserves key characteristics of the donor tumor across successive mouse-to-mouse passages (49). PDXs involve directly implanting patient tumors into nude mice, maintaining the molecular profile of the original tumors, including DNA copy number changes, gene expression, and mutations, which makes them more physiologically relevant compared to traditional cell line models (**Table 1**) (45). The transplantation can be performed either subcutaneously or orthotopically. Subcutaneous models allow for easy tumor formation and size monitoring, while orthotopic models offer a more accurate replication of the native tumor microenvironment, though the procedures are typically more complex (51). Compared to cell line models, PDX models more accurately maintain the genomic integrity and tumor heterogeneity seen in patients (52). However, the PDX models lack a functioning immune system and thus allow evaluating only treatment approaches without involving host immune activation, since T and B lymphocytes, dendritic cells, macrophages, and NK cells are absent. Traditional xenograft models use established cell lines for implantation into animals (53). Due to their indefinite lifespan, easy maintenance, genetic manipulability, and similarity in gene expression to primary human tumors, cell lines are a cost-effective approach to studying cancer (54). These models are used to analyze the impact of genetic modifications or drug treatments on tumor development. Frequently, cell lines are also engineered to express fluorescent or bioluminescent proteins, allowing continuous monitoring of tumor growth through non-invasive imaging techniques. Overall, they serve as an efficient method for studying tumor growth *in vivo* (49).

The PDX model provides several benefits for translational cancer research. The implantation process is minimally invasive for the animal and can be performed using digested segments of actual human tumor samples, enabling the evaluation of various treatments and conditions from a single biopsy (55). Additionally, the implanted tumor can be successfully propagated into new mice for further studies (56). One of the most readily apparent advantages of the PDX model is the ability of tumors in mice to exhibit the same heterogeneity as in humans (57). This enables more specific predictions in clinical models and ensures the validity of the results. In addition, cytogenetic analysis of the models shows that the genetic composition of patients’ tumors is preserved to a high degree, which increases confidence in the model (58). The preservation of histology, gene expression, DNA copy number changes, mutations and response to treatment in studies confirms that the PDX model is a good option for renal cancer research in mice (59). Researchers can rely on their models because tumor growth takes place under physiological conditions, in the presence of oxygen, nutrients and hormones (52). Orthotopic implantation techniques provide the most similar conditions to mimic human conditions, which is beneficial for translational research (49, 60). Overall, this model offers some potential for personalized precision medicine approaches (61).

Although PDX models have proven successful for many studies, they also have disadvantages. One challenge lies in the nature of NOD/scid mice. Due to their suppressed immune system, they are susceptible to thymic lymphomas, which can develop as early as three to four months of age. This can be problematic given their already short lifespan of around eight to nine months. However, it should be noted that male animals develop these tumors to a lesser extent than females and are therefore more suitable for longitudinal studies (61). Furthermore, these mice cannot be used for immunotherapy experiments as they lack an intact immune system (62). Another problem with the PDX model is the low transplantation rate. Approximately only 20% of localized primary tumors are successfully transplanted subcutaneously. When metastatic tissue is transplanted into immunodeficient mice, the success rate for RCC is around 80% (63). Furthermore, the tumors in mouse models may not metastasize in the same way as in patients, which means that some patterns of disease development have been lost (62). A third problem with this model is the loss of the original characteristics of the primary tumor. Heterotopic implantation poses challenges, such as the possibility that the biology of the primary tumor may be compromised (45). Similarly, in this model, the human tumor stroma in the transplanted primary tumor may be replaced by murine stroma after successive passages (63). Although PDX orthotopic models can better mimic the metastatic pattern, they also have their own problems. In general, this more expensive and difficult procedure is challenging (64). RCC are particularly well suited for PDX models (45). The tumors are usually large and rarely treated with chemotherapy, so the behavior and genetics of the mass resemble the original composition and are not affected by exposure to DNA-damaging agents (45, 58). Previous examples of implanted RCCs have shown that the histology and karyotype of patient tumors are preserved (45). Many researchers use PDX models to study the effect of drugs and the sensitivity of tumors to treatments. In addition to surgery, radiotherapy and immunotherapy, molecularly targeted therapy is one of the most important treatment options for patients with kidney cancer (65). There are currently 7 drugs approved for the treatment of all types of kidney cancer (66). Among the best known are sunitinib, everolimus, cabozantinib and temsirolimus. They have all been studied in PDX models (62).

Among the various preclinical models for kidney cancer, PDX models are also a useful model for testing therapeutic approaches for patients. PDX models have been used to study different treatments for kidney cancer. The most commonly used treatment currently is sunitinib. PDX models have been used extensively to study sunitinib, allowing researchers to understand the mechanism of action of sunitinib. Progress has also been made in understanding the effects of sunitinib at the genetic level. The second-line therapy for RCC is everolimus. Everolimus has been studied in mouse models and has been shown to be effective in reducing tumor size. Other drugs that have been studied in PDX models include cabozantinib, temsirolimus and sappanisertib. The use of PDX models enables rapid stratification of different treatments. Another reason for using PDX models is that they can preserve the heterogeneity of the tumor in the mouse (62).

Future models, including humanized mouse models for immunotherapy (67) and mouse avatar models using mice bearing the patient’s tumor, will help determine the optimal choice of chemotherapy for a particular cancer patient (68). In one study, orthotopic mouse models of RCC were first generated and then allogeneic human peripheral blood mononuclear cells were added to investigate the efficacy of an antibody directed against the protein carbonic anhydrase IX in RCC. This study showed that the antibody suppressed cancer growth by stimulating T cell activity (**Table 1**) (46, 69). However, this conclusion can only be considered robust if an isotype-matched control antibody was tested in parallel to fully exclude tumor rejection due to HLA incompatibility or cell-mediated mechanisms unrelated to anti-tumor immunity. Based on available evidence, it remains unclear whether such appropriate controls were included in the referenced studies.

Avatar models can be used for the development of personalized cancer therapies. They enable the production of individualized mouse xenografts and provide a platform for therapeutic decisions (68, 69). In fact, there is technically no difference between an avatar and PDX, but avatars are mostly used in the establishment of personalized medicine to predict the response of specific patients. Gürgen et al. created and characterized a comprehensive panel of PDX models for RCC. What was unique about their cohort is the inclusion of metastases and the development of PDX models from multiple regions for the same patients, reflecting, at least in part, the heterogeneity of RCC tumors. They performed a comprehensive characterization of molecules and drug response. Such a panel can support the development of humanized PDX mouse models for preclinical testing of immune checkpoint inhibitors (70). Humanized mice with an immune system are newly developed animal models. The hematopoiesis of immune-deficient mice is destroyed by irradiating the bone marrow. Hematopoietic stem cells (HSC) derived from humans are then injected into the tail vein or bone marrow cavity to restore a human immune system. The humanized patient-derived xenograft (HU-PDX) model, created by implanting human tumor tissue into humanized mice with reconstituted human immune systems, enables simulation of interactions between human tumors, the human-derived tumor microenvironment, and the human immune system. This approach is only physiologically relevant if both the tumor and the immune system are of human origin; otherwise, if either is murine, cross-species mismatches can limit translational relevance (71–73). The HU-PDX model has great advantages in cancer immunotherapy research and has been used to study colorectal cancer, liver cancer and triple-negative breast cancer (74).

For example, Yubin Kang et al. reported the development of a novel, humanized, patient-derived xenograft model using autologous CD34 cells from bone marrow aspirate of a patient with metastatic clear cell renal cell carcinoma (mRCC), from which a PDX had been developed (**Table 1**) (47).

## 4 Association of TAA and TSA with diseases

Kidney cancer antigens, like antigens from other types of cancer, can interact with the immune response through a variety of mechanisms including activation and inhibition of various components of the immune system. When kidney cancer antigens are recognized by antigen-presenting cells, CD4+ and CD8+ T-cell activation occurs. This can lead to proliferation and differentiation of T cells, which can then attack tumor cells. Also, activation of T cells and other cells of the immune system leads to the release of cytokines that help modulating the immune response to fight tumorigenesis. Therefore, it is important to consider renal cancer antigens as triggers for the development of antitumor immunity in renal cell cancer.

Testicular cancer antigens (CTAs) are tumor-associated proteins that are commonly expressed in normal male germ cells and various types of cancer. The specificity of expression and the highly immunogenic nature of CTAs have made them major potential targets for immunotherapy (75). CT antigens can be divided into classical CT antigens that are encoded on the X chromosome (also referred to as CT-X antigens) and nonclassical (non-X CT antigens) mapping to other chromosomes. Ample knowledge about the *in-situ* presence of several, mostly classical CT antigens such as melanoma-associated antigen-A (MAGE-A), New York esophageal squamous cell carcinoma 1 (NY-ESO-1) has been gathered in recent years (76).

The NY-ESO-1 antigen is capable of inducing an immune response by affecting both humoral and cellular links of immunity, as proven in studies with NY-ESO-1-based vaccines. High antibody titers, strong delayed-type hypersensitivity reactions, and circulating CD8+ and CD4+ T cells specific to NY-ESO-1 have been observed in patients administered these vaccines (77). According to numerous studies of patients with tumors expressing NY-ESO-1, this CTA appears to be the most immunogenic and capable of inducing humoral and cellular immune responses (78).

ENPP3 (ectonucleotide pyrophosphatase/phosphodiesterase may be another, though not specific, target for renal cell carcinoma (RCC) (79). It exhibits substantially higher expression in RCC tumors compared to its limited presence in normal tissues. In the tumor microenvironment, ENPP3 likely drives immunosuppression by modulating nucleotide metabolism and extracellular ATP levels, thereby fostering tumor progression and immune escape (80).

Melanoma antigen-1 (MAGE-1) was the first effective prototype human tumor antigen that caused activation of autologous cytotoxic T lymphocytes in melanoma patients (81). Although MAGE group antigens were first identified in melanoma patients, they were also found in other types of cancer, particularly renal cell cancer (82). In a study by Alma Demirovic et al, the expression of MAGE-A and NY-ESO-1 antigens in renal oncocytoma and chromophobe renal cell cancer was studied by immunohistochemistry. Of the 17 renal oncocytoma samples, 88.2% of the samples showed positive staining for both antigens, and 38.9% and 33.3% of the 18 ChRCC samples, respectively (83).

Another widely investigated target CTA for immunotherapeutic strategies is Preferentially Expressed Antigen in Melanoma (PRAME). This melanoma-associated antigen, which belongs to non-classical CTAs, is also expressed in various neoplasms and normal adult tissues, including kidney cancer (84). PRAME suppresses T-cell activation and cytolytic potential; accordingly, exposure to PRAME antigen improves T-cell activation and cytotoxicity (85). In the study, monoclonal antibodies were used for immune-histochemical detection of PRAME in various tumor and healthy tissue samples. Expression of PRAME protein in renal cell carcinoma samples were positive in about one-fifth of the luminal cell cases tested (76). This antigen is highly expressed in melanoma (86), leukemia (87), ovarian cancer (88), breast (85), cervical (89), lung (90), and sarcoma (91).

CTA expression has been demonstrated in many other varieties of human tumors: esophageal cancer (92), penile cancer (93), melanoma (94), non-small cell lung cancer (95), hepatocellular carcinoma (96), and breast cancer (97). Many clinical trials are currently underway. The vast number of CTAs are expected to provide a pool of antigenic peptides that will induce humoral or cell-mediated immune responses in the human host that target tumor cells (98).

The hypoxic tumor microenvironment makes cancer cells resistant to therapy. HIF, which may result from mutations in the VHL gene, play a key role in this process (99). HIF-1α promotes cancer development by stimulating angiogenesis, cell division, survival, metabolic alteration, invasion, metastasis, and maintenance of cancer stem cells. It also induces genetic instability and treatment resistance. HIF-1α is activated not only by hypoxia but also through other signaling pathways (100). Several studies have shown that HIF-1α expression is upregulated in many types of cancer, such as kidney cancer, gastric cancer (101), colorectal cancer (102), papillary thyroid cancer (103), non-small cell lung cancer. Renal cell cancer is also frequently associated with mutations in the VHL gene. HIF-1α peptides can induce cytotoxic T lymphocytes in patients with renal cell carcinoma, which opens the possibility of creating anti-cancer vaccines (104).

Carbonic Anhydrase IX (CAIX) is a surface protein that is activated by hypoxia and is often associated with renal cancer (105). The results of a study by Arsani Y. et al. confirm that intense CAIX staining is characteristic of renal cell cancer and is expressed in 94% of RCC samples tested (106). *In vitro*, various mechanisms of immune response to CAIX antigen were tested and revealed that anti-CAIX monoclonal antibodies were able to induce immune killing of renal cell cancer, including NK cell cytotoxicity, complement-dependent cytotoxicity and macrophage phagocytosis. Moreover, the expression level of CAIX on tumor cells correlates with the activity of the immune response (46). CAIX is normally expressed in the mucosa of the stomach, duodenum and bile ducts (107). Some tumors such as ccRCC, glioblastoma, triple negative breast cancer, ovarian cancer, colorectal cancer and others and others also overexpress carboanhydrase IX isoform (CAIX).

B7-H3, also known as CD276, belongs to the B7 family of proteins that regulate the immune system (108). B7-H3 is overexpressed in many solid tumors, including prostate cancer, renal cell carcinoma, melanoma, head and neck squamous cell carcinoma, non-small cell lung cancer, and breast cancer (109). The ability of B7-H3 to inhibit the proliferation of CD4^+^ and CD8^+^ T cells has been discovered (110). Targeting this antigen with monoclonal antibodies also helps to combat cancer cells through antibody-dependent cytotoxicity (111).

5T4 trophoblast glycoprotein (5T4) is a secreted membrane glycoprotein expressed on clear cell and papillary renal cell carcinomas. Immunohistochemistry of frozen sections established that the monoclonal antibody 5T4 detects expression of the antigen by many different types of carcinoma such as gastric, colorectal, and ovarian carcinomas, but shows low levels of expression in some normal adult tissue epithelia. The antigen appears to be expressed by a somewhat specialized epithelium, as it is not detected in adult liver, bronchial, heart, testicular, ovarian, brain, and muscle tissues, but is strongly expressed by syncytotrophoblasts, some extravillous cytotrophoblasts, and amniotic epithelium (112). 5T4 is involved in antibody-dependent cell-mediated cytotoxicity because it is not released from the cell membrane. The antigen can also induce cellular immunity because 5T4-transduced cell lines are recognized by T lymphocytes *in vitro*. In addition, 5T4-transfected tumor cells exhibit altered morphology and increased motility, which plays a role in tumor progression and invasion. 5T4 expression in colorectal, gastric and ovarian cancers has been shown to correlate with poorer clinical prognosis, confirming the role of the antigen in tumor development and spread (113). Immunization with recombinant modified cowpox virus Ankara encoding human 5T4 (MVA-5T4) resulted in 5T4-specific antibody formation and T-cell immune response. Thus, MVA-5T4 vaccination generates 5T4-specific humoral and cell-mediated immunity that, when administered with standard doses of IL-2, improves therapeutic responses (112).

Tumor-associated antigens of clear cell renal cell cancer include Mucin-1 (MUC-1) antigen, a transmembrane glycoprotein expressed by the MUC-1 gene on endothelial cells and in cells associated with renal development. In normal tissues, the protein is expressed on the apical surface of glandular cells in breast, lung, pancreas, kidney, female reproductive tract and stomach (114). Aberrant expression of MUC1 occurs in cancer cells of esophagus, stomach, breast cancer, ovarian cancer, bladder cancer and other tumors (115). During malignant transformation of MUC-positive cells, there is an increase in expression, changes in cell distribution, nature and degree of glycosylation (116). Aberrantly glycosylated MUC1 is a recognized tumor-specific antigen on epithelial cell tumors (117). Several HLA-A*02-restricted peptides have been derived from MUC-1, which *in vitro* induce a T-cell response capable of killing MUC1-expressing cells (including squamous cell cancer cell lines) (118). Numerous therapeutic approaches to target MUC1 have been investigated, including vaccination with a modified cowpox virus expressing IL-2 and MUC1 and a TAA peptide vaccine incorporating peptides targeting MUC1 (119).

CD70 is a protein encoded by the CD70 gene in humans. It acts as a costimulatory molecule, improves the activation, proliferation and survival of T cells and B cells. It is mainly expressed on highly active T cells and B cells, but also on NK cells and mature dendritic cells. In oncology, CD70 is aberrantly expressed on malignant cells without (solid tumors) or with CD27 co-expression (hematologic malignancies), promoting immune evasion through the tumor microenvironment and tumor progression (120). CD70 overexpression has been reported in renal cell carcinoma, Hodgkin’s lymphoma, non-Hodgkin’s lymphoma. The antigen evades immunity by inducing cytotoxic action on B- and T-lymphocytes. CD70 expression was even higher in metastatic samples of lung carcinoma, pancreatic carcinoma, and osteosarcoma, suggesting the importance of CD70 in disease progression (121). The aforementioned and additional antigens and their associations with cancer are presented in **Table 2**.

**Table 2:** Association of tumor antigens with immune response and related diseases.

Antigen	Coding gene	Human/Cell line/animal	Recognition by T - lymphocytes	Other diseases associated	Healthy tissue	Ref.
RU2AS	Antisense transcript of RU2	Renal carcinoma cell lines	+	Nephronophthisis 19 and Isolated Neonatal Sclerosing Cholangitis.	Testis and kidney, and, at lower levels, in urinary bladder and liver.	(122)
ENPP3	ENPP3 gene	Renal carcinoma cell lines	+	liver, renal, and colorectal carcinomas	Cervix, intestine, small intestine	(80, 123)
MAGE-A3/4	MAGEA3 Gene	ChRCCs cell lines	+	Melanoma, Testicular Cancer, Squamous Cell Carcinoma, advanced gastric carcinomas and hepatocellular carcinomas	Testicular germ cells	(83)
PRAME		Renal cell carcinoma, clear cell	+	Carcinomas of endometrial, serous ovarian, mammary ductal, lung, and renal	Testicular germ cells, Endometrium, cycling, Lymph node	(76)
Hypoxia-inducing factor (HIF)-1α	Mutated Von Hippel Landau (VHL) gene	l RCC cell lines	+	Pheochromocytoma, Enchondromatosis, Prostate Cancer		(104)
Nes2LR		Adenocarcinoma RENCA cancer model				(124)
CAIX	Gene CAIX	Renal cancer cell line (Renca)	+	Renal Cell Carcinoma	Small amount of hCAIX expression in gastric mucosa and bile duct epithelial cells.	(46, 125, 126)
B7H3 (CD276)	Gene B7H3	Tumor cell lines	+	Prostate cancer, renal cell carcinoma, melanoma, squamous cell carcinoma of the head and neck, non-small cell lung cancer, and breast cancer	Liver, urothelium, and fetal kidney.	(109)
RAGE-1	Gene RAGE-1	Renal cell carcinoma cell line LE9211-RCC of human	+	Sarcomas, bladder tumors, and melanomas	Retina	(127)
HMGB1			+	Melanoma, nasopharynx cancer, breast cancer, colorectal cancer, cervical cancer and bladder cancer		(109)
CD70	CD70	Renal cell carcinoma	+	Solid tumors, including brain tumors, RCC, thymic carcinoma, nasopharyngeal carcinoma, ovarian, lung, colon and pancreatic cancer, and melanoma	Due to the aberrant up-regulation of CD70 in RCC cells, its membrane surface expression, its limited, normal tissue distribution ()?	(128)
Mucin-1	MUC1	Clear-cell renal-cell carcinoma	+	In cancer cells, including esophageal cancer, stomach cancer, breast cancer, ovarian cancer, bladder cancer and other tumors, especially noticeable in breast cancer cells	Apical surface of most glandular epithelial cells, including those of the mammary gland, lung, pancreas, kidney, female reproductive tract and stomach	(129)
Protein melan-A	MLANA	Clear-cell renal-cell carcinoma	+	Perivascular epithelioid cell tumors (angiomyolipoma, lymphangioleiomyomatosis and clear cell tumor)	Specific for the melanocyte lineage, found in normal skin, the retina, and melanocytes, but not in other normal tissues.	(130)
5T4 Trophoblastglycoprotein	5T4	Clear-cell renal-cell carcinoma	+	Colorectal, Gastric and ovarian cancers	Expressed only by the syncytiotrophoblast, some extravillous cytotrophoblast and the amniotic epithelium	(15, 112)

No data could be found for the empty fields.

Thus, antigen-directed therapy may be an important component in the treatment of renal cell carcinoma by various immunization strategies.

## 5 Review of current clinical trials based on TSAs and TAAs

Already in 2004 a clinical phase III study was published reporting the effects of an adjuvant autologous renal tumor cell vaccine (i.e., lysate from autologous tumor cells which had been incubated with interferon γ) conducted in patients with renal cell carcinoma after radical nephrectomy (131). This multicenter study investigating the risk of tumor progression was carried out in 558 patients of whom 379 were eligible for assessment. Tumor progression was determined 60 months and 70 months after treatment in vaccinated patients versus an untreated control group. Vaccination was well tolerated with a clear beneficial effect for the vaccinated group (131).

Furthermore, over the past few years, significant changes in the treatment patterns of patients with renal cancer have been reported with the introduction of immunotherapeutic agents (132). Cancer immunotherapy is a promising strategy for the treatment of the disease. However, immunotherapy is not widely used in renal cell cancer because only a few patients show a positive response (33). More recently, the combination of immunotherapeutic agents with anti-angiogenic agents has proven to be an effective therapeutic strategy (13). Despite the availability of potentially useful TSAs and TAAs as therapeutic agents for the treatment of RCC, only a few clinical trials have been conducted so far, most of which are still in the early stages (**Table 3**).

**Table 3:** Review of clinical trials based mainly on specific TSA/TAA peptide vaccines in RCC therapy.

Vaccine name	Brief description	Results	Ref.
IMA901	Vaccine contains 10 different peptides corresponding to tumor antigens of most renal cell carcinomas: NOX1, FERMT1, ODC1, PCNA, TGFB1, TOP2A, TOP2B, CEACAM5, CCND1, MUC1, MMP7, MET.	In the Phase 1 study of IMA901 plus GM-CSF in 20 (74%) of 27 patients, a T-cell response to at least one tumor-associated peptide was observed. In the Phase 2 trial (NCT00523159), a T-cell response was reported in 39 (64%) patients, including 16 (26%) who responded to more than one tumor-associated peptide.	(133, 134)
TG4010	A cancer vaccine based on a modified vaccinia virus expressing MUC1 and interleukin-2, in combination with cytokines, as first-line therapy in metastatic RCC.	5/28 evaluable patients developed a MUC1-specific CD4+ T-cell response during therapy, and 6/23 developed a MUC1-specific CD8+ T-cell response before or during therapy. However, there were no objective responses to therapy	(119)
Dendritic cell-based vaccina	Autologous mature dendritic cells derived from peripheral blood monocytes were pulsed with the HLA-A2-binding MUC1 peptides (M1.1 and M1.2). For the activation of CD4(+) T-helper lymphocytes, dendritic cells were further incubated with the PAN-DR-binding peptide PADRE.	Twelve of 18 patients showed a response to the MUC1 peptide in CTL, and 10/18 had a PADRE-specific response. Four more patients remained stable for 14 months.	(135)
Vaccination of CA9-derived peptides	The vaccine includes synthetic peptides corresponding to three putative CAIX T-cell epitopes (CA9p219-227, p288-296, and p323-331)	Cytolytic T-cell reactivity specific for one or more peptides was observed in 76% of patients.	(136)
Human vascular endothelial growth factor receptor 1 peptide vaccines	The vaccine is a peptide derived from VEGFR1 restricted to HLA-A*0201 or HLA-A *2402	Of the 18 patients, 2 had a partial response to treatment. Stable condition for more than 5 months was observed in 8 patients with a median duration of 16.5 months (4–32 months).	(137)
Naptumomab Estafenatox + IFNα versus IFNα	The vaccine contains a superantigen, naptumomab estafenatox (5T4FabV18-SEA/E-120 or ABR-217620), that targets tumors.	Median OS/PFS for Nap + IFN patients was 17.1/5.8 months versus 17.5/5.8 months for the patients receiving IFN alone.	(138)
HIG2-9-4	A highly immunogenic peptide epitope (HIG2-9-4) restricted to HLA-A*0201/0206 corresponding to a portion of HIG2 is used as a therapeutic vaccine	Peptide-specific cytotoxic T lymphocyte (CTL) responses were detected in eight of the nine patients.	(139)
CTT1057	CTT1057 is a small-molecule inhibitor of PSMA	The results have not been published.	NCT03427476
CAR-T/TCR-T-cell immunotherapy	Multitarget gene-modified immunotherapy using CAR-T/TCR-T cells incorporating ten different tumor-specific antibodies	The results have not been published.	NCT03638206
Montanide ISA-51	Synthetic adjuvant peptide in combination with immune adjuvants (granulocyte-macrophage colony-stimulating factor; ISA-51 montanide)	The results have not been published.	NCT02429440
FLT3L with melanoma-associated peptides	Recombinant FLT3L alone or with melanoma-associated peptides (MART-1, gp100:209-217, gp100:280–288 and tyrosinase)	Status unknown	NCT00019396
WT1	Anchor modified, HLA-A*2402 binding WT1 peptide which was emulsified in Freund’s incomplete adjuvant.	In 2/3 patients tumor growth was suppressed and clinical response was evaluated as stable disease by the RECIST criteria after 3 months of weekly immunizations.	(140)
WT1 peptide-loaded dendritic cells	The study was conducted by vaccination with WT1 peptide-loaded dendritic cells (DCs) and OK-432 adjuvant combined with molecular targeted therapy or conventional chemotherapy.	Seven of the ten patients (5 with RCC and 5 with BC) had a stable disease course, and three had disease progression after vaccination with WT1 peptide-loaded dendritic cells	(141)

IMA901 is a therapeutic renal cell carcinoma vaccine containing 10 different synthetic peptides derived from tumor antigens of most types of renal cell carcinoma: NOX1, FERMT1, ODC1, PCNA, TGFB1, TOP2A, TOP2B, CEACAM5, CCND1, MUC1, MMP7, MET. Clinical trials of the IMA901 vaccine were conducted in HLA-A*02-naïve kidney cancer patients. Prior to vaccination, patients were treated with cyclophosphamide to reduce regulatory T cells. Vaccination induced a T-cell response specific for kidney cancer antigens. In a phase 1 study of IMA901 plus granulocyte-macrophage colony-stimulating factor (GM-CSF) in 30 patients with metastatic renal cell carcinoma, in 18 the combination was shown to be safe, and in 20 (74%) of 27 patients, a T-cell response to at least one tumor-associated peptide was observed. In a subsequent randomized phase (NCT00523159), 68 patients with relapsed or refractory renal cell carcinoma received IMA901 plus GM-CSF with or without a single infusion of cyclophosphamide (300 mg/m²). T-cell responses were reported in 39 (64%) patients, including 16 (26%) who responded to more than one tumor-associated peptide. During Phase 3 (NCT01265901), it was found that IMA901 did not improve overall survival when added to sunitinib as first-line therapy in patients with metastatic renal cell carcinoma (133, 134). The MVA vector-based cancer vaccine TG4010 targeting the MUC1 antigen was tested in a multicenter phase II trial in renal cell carcinoma. Thirty-seven patients with advanced MUC1-positive PCR received TG4010 at 10 (8) units/injection weekly for 6 weeks, then every 3 weeks until progression, when TG4010 was continued in combination with interferon-α2a and interleukin-2. Five out of 28 evaluable patients developed a MUC1-specific CD4+ T-cell response during therapy and 6/23 developed MUC1-specific CD8+ T-cell responses before or during therapy. However, there were no objective responses to therapy (119). A phase I trial was conducted to evaluate the feasibility, safety and efficacy of dendritic cell-based vaccination in patients with metastatic renal cell carcinoma. Autologous mature dendritic cells derived from peripheral blood monocytes were pulsed with HLA-A2-binding peptides MUC1 (M1.1 and M1.2). To activate CD4+ T helper lymphocytes, dendritic cells were additionally incubated with the PAN-DR-binding peptide PADRE (142). The study included 20 patients with metastatic renal cancer HLA-A2+ tumors expressing MUC1, dendritic cell vaccination was administered intravenously every 2 weeks for four times and repeated monthly until tumor progression. After five dendritic cell injections, patients additionally received three weekly injections of low-dose interleukin-2 (1 million IU/m2). Twelve of the 18 patients studied developed a detectable response to the cytotoxic T lymphocyte (CTL) peptide MUC1, and 10/18 had PADRE-specific responses. Three objective responses were observed, including one complete response (135).

A phase l clinical trial was conducted in which synthetic peptides corresponding to three putative T-cell epitopes of CAIX (CA9p219-227, p288-296, and p323-331) were used. Twenty-three patients positive for HLA-A24 and histologically confirmed RCC were included in the study. After 6–9 intradermal doses of the vaccine, cytolytic T-cell reactivity specific for one or more peptides was observed in 76% of patients. Three patients with multiple lung metastases showed a partial response with disappearance and reduction in the size of metastatic lesions. These results suggest that vaccination with these peptides is safe and may be applicable for patients with cytokine-refractory renal cancer (136). In a phase I clinical trial to evaluate the safety of vaccination with vascular endothelial growth factor receptor 1 (VEGFR1) peptide, 18 patients were subcutaneously injected with a VEGFR1-derived peptide restricted to HLA-A*0201 or HLA-A *2402 weekly for 5 weeks and then every 2 weeks thereafter. Of the 18 patients, 2 had a partial response to treatment. A stable condition for more than 5 months was observed in 8 patients with a median duration of 16.5 months (4–32 months). The proven safety of the vaccine warrants further studies (137).

A novel tumor-targeted superantigen, naptumomab estafenatox (5T4FabV18-SEA/E-120 or ABR-217620), was developed based on experience from clinical trials of superantigen preparations. Naptumomab estafenatox is a fusion protein that conjugates a variant of a bacterial superantigen to the Fab-binding domain of the 5T4-specific monoclonal antibody to activate both CD4+ and CD8+ T cells at 5T4-expressing tumors (143). In a phase II/III study, 512 patients with renal cancer were randomized to receive naptumomab estafenatox with IFN-α or IFN-α alone (NCT00420888).This trial failed to meet the primary endpoint of improved survival for patients receiving naptumomab estafenatox (OR 1.08; P = 0.56). At this time, the trial has been terminated (144, 145).

In a dose escalation phase I study, a HIG2-9–4 peptide restricted to HLA-A *0201/0206 was used to vaccinate nine patients with refractory mRCC after failed therapy with cytokines and/or tyrosine kinase inhibitors. The vaccine was administered subcutaneously weekly for 4 weeks in each cycle, and vaccination cycles were continued until disease progression. HIG2-9-4- specific responses to CTL were detected in eight of nine patients; however, due to the small number of patients, it is difficult to draw definite conclusions (139).

Another study used CTT1057, a small-molecule inhibitor of Prostate Specific Membrane Antigen (PSMA), as a novel imaging agent for neovascularization in renal cell cancer (NCT03427476). The agent binds PSMA and is designed to detect tumors expressing PSMA, such as has been described for some RCC tumors. Because of its irreversible binding to PSMA and rapid uptake by PSMA-expressing cancer cells, accumulation in the cancer target is expected to be rapid, specific and sensitive. PSMA expression has been reported in renal cell carcinoma cells, making CTT1057 feasible for the detection of these tumors. The aforementioned study enrolled 10 patients and the study has now been completed but the results have not been published.

An open-label study was also conducted to evaluate the safety and efficacy of chimeric antigen receptor T-cell (CAR-T)/TCR-T-cell immunotherapy for the treatment of various malignancies in patients, including kidney cancer (NCT03638206). The study is a multi-targeted gene-modified immunotherapy using CAR-T/TCR-T cells which include ten different tumor-specific antibodies, anti-C-met antibody immunotherapy was planned for the therapy of patients with renal carcinoma. Patients were planned to receive CAR-T-cell immunotherapy with several different specific chimeric antigenic receptors targeting different antigens, respectively, by infusion. The status of the study is unknown at this time.

A randomized trial targeting patients with renal cell cancer of clinical stage T3 or T4, N0, M0 renal cell cancer has been initiated at the University Hospital Tuebingen, Germany (NCT02429440). Adjuvant antigen-specific immunotherapy is planned for the treatment of patients with metastatic renal cell cancer using tumor-associated peptides. The study investigated the feasibility and tolerability of immunization with a synthetic adjuvant peptide in combination with immune adjuvants (granulocyte-macrophage colony-stimulating factor; ISA-51 montanide) in patients with metastatic RCC. The status of the clinical trial is unknown at this time.

A Phase II study was conducted to investigate the efficacy of Flt3L alone and in combination with immunization with melanoma peptides (MART-1, gp100:209-217, gp100:280–288 and tyrosinase) in the treatment of patients with metastatic melanoma or RCC (NCT00019396). The Flt3L drug can stimulate the human immune system and promote the destruction of tumor cells. Vaccines made from melanoma cells may induce an immune response to tumor cells and eliminate them. Participants are divided into 3 cohorts, one of which includes patients with RCC. The trial has now been completed but the results have not been published.

Tumor-specific immunotherapy with Wilms’ tumor 1 (WT1) peptide has been clinically tested in leukemia, myelodysplastic syndrome, breast and lung cancer and is showing some promising results. In this study, three patients with renal cell cancer were treated with a modified anchor, HLA-A* 2402-binding peptide WT1, which was emulsified in Freund’s incomplete adjuvant. In two patients, tumor growth was suppressed and clinical response was graded as stable disease according to RECIST criteria after 3 months of weekly immunizations (140).

In a phase I/II study, 5 patients with metastatic renal cancer and 5 patients with metastatic bladder cancer were treated with DCs conjugated to WT1 peptide in combination with adjuvant OK-432 in combination with molecular targeting therapy or conventional chemotherapy. Seven patients showed a stable disease course, while three patients showed disease progression after DC vaccination. DC vaccination enhanced WT1-specific immunity and decreased regulatory T cells, which may be associated with clinical outcome (141).

Patient enrollment is underway for a phase 1 open-label, dose escalating, extended cohort study of P-MUC1C-ALLO1 in adult patients with advanced or metastatic solid tumors, including renal cancer (NCT05239143). P-MUC1C-ALLO1 is an allogeneic CAR-T therapy designed to target cancer cells expressing the C-terminal cell surface-associated antigen Mucin1 (MUC1-C). Additional participants will receive treatment with P-MUC1C-ALLO1 at a defined recommended Phase 2 dose (RP2D).

Despite several ongoing studies, there has been no definitive positive experience with TSA- and TAA-peptide based vaccines for the treatment of RCC at this time. The results of the aforementioned clinical trials are summarized in **Table 3**.

Accordingly, there are currently no approved tumor-specific vaccines or other antigen-specific compounds for the treatment of disseminated RCC.

## 6 An overview of vaccine approaches for RCC and considerations from outside the field of oncology

While for certain forms of cancer preventive forms of vaccination have emerged (31, 146, 147), current approaches for RCC mainly focus on therapeutic vaccination strategies based on TSAs such as neoantigens (27, 148), or TAAs (148). Neoantigens are newly formed antigens made by tumor cells by several mechanisms such as mutations, altered RNA splicing or reading frames, changed post-translational modifications or integrated viral sequences (27, 148). As a result, TSAs are highly specific for the tumor, naïve to the immune system and usually per se immunogenic. By contrast, TAAs may occur not only in various tumors but also in normal tissues and therefore are less specific for certain tumors und poorly immunogenic because of established central or peripheral immune tolerance. TSAs are usually immunogenic but expressed often only in certain tumor types or only in tumors from certain patients and hence require personalized treatment approaches (19). The advantage of TAAs is that they are expressed not only in different tumors but also in different patients at high rates and may therefore be considered as frequent target antigens requiring a less personalized treatment approach. Regarding TAAs, the low immunogenicity of the target antigen represents a major challenge for vaccination because due to central tolerance TAA-specific T cells have been largely eliminated (18). Accordingly, poor or no activation of TAA-specific CD4^+^ help for antibody production and poor or no generation of TAA-specific cytotoxic CD8^+^ T cells can be expected upon vaccination with TAAs regardless of what vaccination strategy (e.g., protein-based, peptide-based or genetic vaccine) is chosen.

Can there be something learned from other forms of therapeutic vaccination? In fact, regarding therapeutic vaccination in allergy it has been found that a reduction of allergen-specific T cell epitopes is important to reduce T cell-mediated side effects in recombinant allergen-based vaccines (149, 150). Accordingly, B cell epitope-based allergy vaccines were made which not only lack immunoglobulin E (IgE) recognition of anaphylactogenic epitopes capable of inducing immediate type allergic reactions but also allergen-derived T cell epitopes with the goal of inducing allergen-specific IgG antibodies (144). In order to render the allergen-derived B cell epitope-containing peptides immunogenic they were fused to an immunological carrier protein. This new class of therapeutic fusion protein-based allergy vaccines was shown to induce robust allergen-specific IgG antibodies which protected allergic patients from allergen-induced symptoms (145). A similar approach has been used also for cancer vaccination by coupling Her-2/neu peptide mimotopes to an unrelated carrier protein. Antibodies induced by this vaccine were found to inhibit tumor growth in an *in vivo* model (151). Likewise, work regarding MUC1 by Olivera Finn showed that during tumor transformation, abnormal (shortened) glycosylation of MUC1 exposes the epitopes of the protein core, making them more immunogenic and accessible to T-cell recognition. Conjugation of such MUC1 epitopes with an immunogenic KLH carrier in the presence of adjuvants (e.g., QS-21) enhances T-helper assistance and induces MUC1-specific antibodies and T-cell responses, which has been shown in preclinical studies and early clinical trials involving patients (including breast carcinomas) (152–154).

The use of carrier proteins has been also used recently for the development of SARS-CoV-2 vaccines in order to render SARS-CoV-2 antigens, in particular the receptor-binding domain RBD, more immunogenic and to overcome immunological non-responsiveness (155).

**Figure 1:**
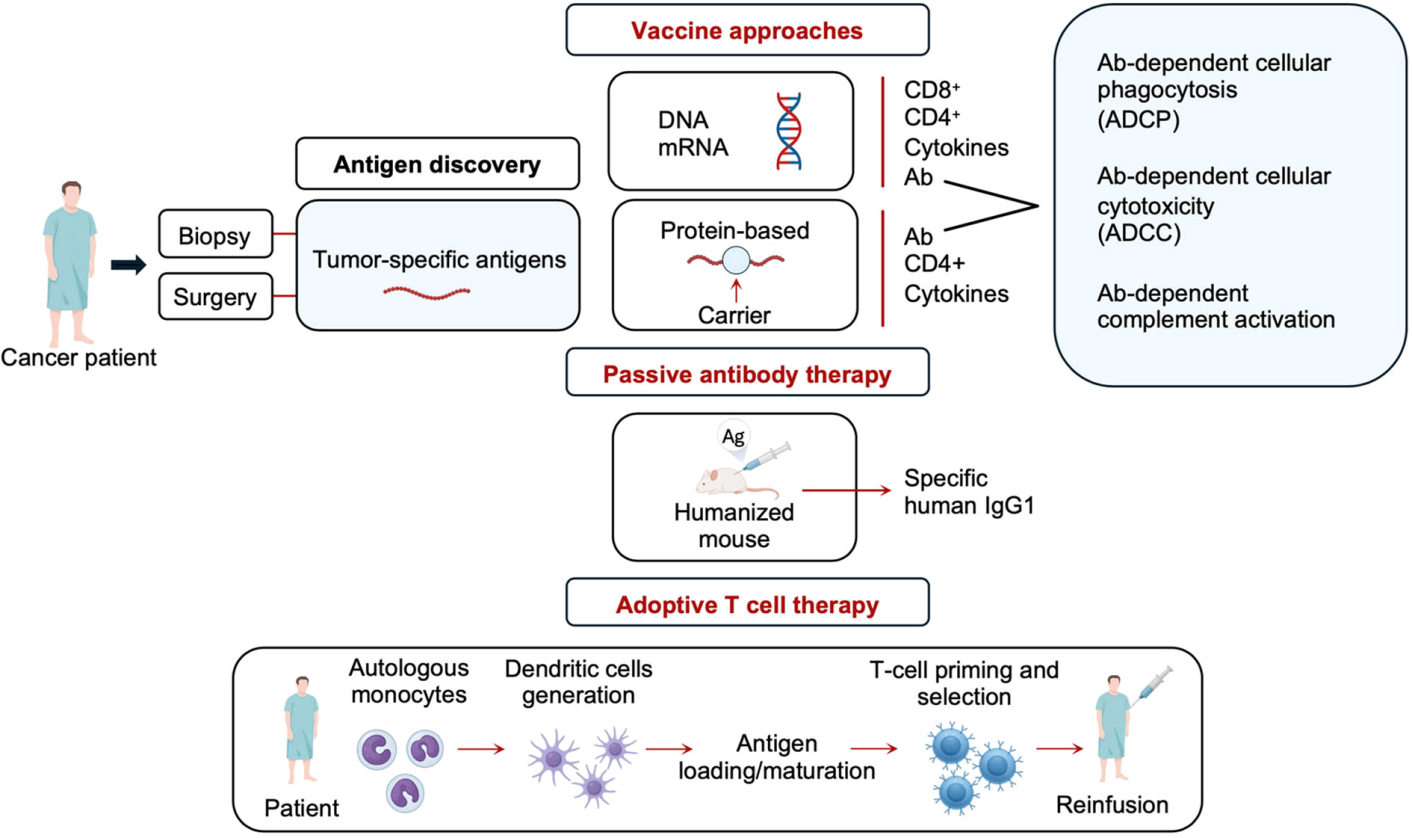
Possible approaches for antigen-specific treatments of cancer.

Accordingly, the idea of proposing TAA-carrier-based vaccines is that carrier-specific T cell help may support the production of TAA-specific antibodies and thus overcome central T cell tolerance against TAAs. In fact, such an approach has been used also in the field of allergy to overcome tolerance towards IgE. IgE is a molecule known to the immune system of the host so that it is difficult to induce anti-IgE antibodies by active vaccination. In order to induce therapeutic anti-IgE antibodies, the approach was taken to fuse portions of the IgE constant region to a carrier protein. This would induce IgG antibodies which would block IgE binding to its receptors and thus IgE-mediated inflammation (156–158). From the naïve view of allergologists and immunologists having worked on the development of therapeutic allergy vaccines, it is hypothesized that one may be able to develop carrier-based TAA vaccines for their ability to induce TAA-specific antibodies. Clearly such vaccines will induce mainly TAA-specific antibody responses, whereas the expectation that TAA-specific T-cell responses can be recruited is rather low. However, tumor-specific antibody-based treatments are effective because antibodies can induce several tumor-killing effects such as antibody-dependent activation of NK cells, phagocytosis and complement (31) (**Figure 1**).

Due to the availability of differential transcriptomics technologies which allow a fast identification of TSAs and TAAs by biopsy and after surgery, the discovery of tumor antigens will be greatly advanced in the future. The development of tumor antigen-specific treatment approaches has likewise accelerated. Both genetic and protein-based vaccines can be rapidly developed even in a personalized manner to have vaccines available for prevention of metastasis after surgery (**Figure 1**, left). It is even possible that tumor antigen-specific human monoclonal antibodies can be rapidly developed because one may use “humanized mice” to generate human monoclonal antibodies relatively quickly (159–161). The effectiveness of monoclonal antibody-based treatment strategies for cancer is well established (162–166) but has not been applied for RCC.

Likewise, peptide-based vaccines can be rapidly developed because synthetic peptide chemistry and algorithms for major histocompatibility complex (MHC) matching are well developed. Peptides can be administered directly to the patient or used to load patients derived dendritic cells or to expand patients-derived cytotoxic T cells and reinfuse them to the host (**Figure 1**) (167–169).

In recent years, genetic vaccines based on DNA and mRNA technologies have attracted the attention of oncologists. Their main advantage that this technology allows to quickly develop and produce patient-specific vaccines that encode selected tumor or neoantigens. Several early clinical trials have demonstrated the feasibility and immunogenicity of personalized mRNA vaccines for solid tumors, including RCC and melanoma. These vaccines can induce both a cytotoxic and a helper CD8^+^ T-cell response and can be adapted to individual mutation profiles (170).

In addition to their flexibility and rapid development time, DNA and mRNA vaccines allow for the inclusion of multiple antigens in a single construct, thereby improving immune system recognition and reducing the risk of immune-mediated tumor escape (171). Unlike traditional peptide or protein vaccines, genetic vaccines do not require large-scale antigen production and purification, which simplifies manufacturing and facilitates rapid clinical translation (172).

However, the immunogenicity of genetic vaccines depends critically on the delivery platform and intracellular translation efficiency. Optimization of lipid nanoparticle formulations, vector backbones, and codon usage has proven crucial for achieving robust antigen expression and effective T-cell priming (173).

Taken together, nucleic acid–based vaccines represent a conceptual step forward toward individualized immunotherapy, linking advances in genomics, antigen discovery, and vaccine engineering to clinical cancer treatment. **Table 4** outlines possible strengths and limitations of several tumor antigen–specific treatment approaches – including peptide, protein (carrier-fusion), genetic (DNA/mRNA), and antibody-based approaches.

**Table 4:** Characteristics, possible advantages and disadvantages of tumor antigen-specific treatment strategies.

Characteristics	Possible advantages	Possible disadvantages
Recombinant tumor antigen made in different expression systems combined with different adjuvants	Can induce tumor antigen-specific CD4+, cytokine and antibody responses Can be quickly made by synthetic gene technology and subsequent expression Can be precisely dosed Adjuvant can direct immune response and capture antigen at the injection site	Limited induction of antigen-specific CD8+ responses Poor immunogenicity for TAAs
Synthetic tumor antigen-derived peptides activating CD4+ and/or CD8+ T cells	Peptides are easy to make Can induce cytotoxic CD8+ responses	Cannot induce antigen-specific antibodies Poor immunogenicity for TAAs Limited to certain patients by MHC restriction
Recombinant carrier-fused tumor antigen made in different expression systems combined with different adjuvants	Can induce potent tumor antigen-specific antibody responses High immunogenicity, can overcome tolerance due to carrier-specific T cell help Can be quickly made by synthetic gene technology and subsequent expression Can be precisely dosed Adjuvant can direct immune response and capture antigen at the injection site	Limited induction of antigen-specific CD4+, cytokine and CD8+ responses
DNA or RNA encoding tumor antigens delivered to host cells to produce the corresponding tumor antigen	Can induce tumor antigen-specific CD4+, cytokine, CD8+ and antibody responses Can be quickly made without need to produce the tumor antigen by recombinant means	Cannot be precisely dosed Produces circulating antigens which may form immune complexes leading eventually to Type III immune pathology CD8+ cells may attack normal host cells producing the tumor antigen in the course of repeated vaccination-risk of inducing autoimmunity May induce side effects which are uncommon for protein-based vaccines Poor immunogenicity for TAAs
DNA or RNA encoding carrier-fused tumor antigens delivered to host cells to produce the corresponding tumor antigen	Can induce tumor antigen-specific CD4+, cytokine, CD8+ and antibody responses Can be quickly made without need to produce the tumor antigen by recombinant means High immunogenicity because it can overcome tolerance due to carrier-specific CD4+ T cell help	Cannot be precisely dosed Produces circulating antigens which may form immune complexes leading eventually to Type III immune pathology CD8+ cells may attack normal host cells producing the tumor antigen in the course of repeated vaccination-risk of inducing autoimmunity May induce side effects which are uncommon for protein-based vaccines
Recombinant human tumor-antigen-specific antibodies which are engineered for different purposes	Can precisely bind and attack tumor cells Can be rendered toxic for tumor cells using for example toxins, radionuclides and can attract T cells or built into CAR cells	*Per se* and without engineering cannot elicit anti-tumor T cell reactivity

Synthetic peptide vaccines and recombinant protein vaccines are well-established and technically straightforward to produce. They allow precise control over antigen dose and enable modulation of immune responses by selecting appropriate adjuvants and delivery routes. Carrier-based protein vaccines, in which tumor antigens are fused to an immunogenic carrier (e.g., viral or bacterial proteins), may enhance antigen presentation through MHC class II, promoting CD4^+^ T cell activation via the carrier and antibody production against both, the carrier and tumor antigen.

However, insufficient induction of CD8+ cytotoxic responses against the tumor antigen may be a limitation. Additionally, many tumor-associated antigens are self-antigens, making it difficult to overcome central and peripheral tolerance. Therefore, adjuvants that stimulate Th1-polarized immunity (such as saponins or toll-like receptor (TLR) agonists) are necessary to enhance cellular responses and promote antibody formation capable of mediating antibody-dependent immune activation. However, there is a possibility to induce also CD8^+^ T cell responses by subunit vaccines via cross-presentation. Cross-presentation is a central immunological mechanism that greatly influences the effectiveness of cancer subunit vaccines. This mechanism is particularly important for vaccines that contain purified tumor antigens, such as peptides, proteins, or neoantigens. Unlike live pathogen-based vaccines, subunit vaccine antigens do not naturally enter the MHC class I pathway, which is required to activate CD8^+^ cytotoxic T lymphocytes, the main effector cells responsible for killing tumor cells.

Cross-presentation is an unusual but essential mechanism for anti-tumor immunity. It refers to the ability of specialized antigen-presenting cells – primarily dendritic cells, and to a lesser extent M1 macrophages – to take up exogenous antigens (174), process them into peptides, load them onto MHC class I molecules (normally reserved for endogenous antigens), and prime naïve CD8^+^ T cells. This process generates tumor-specific CTLs capable of recognizing and killing cancer cells. This mechanism leads to clonal expansion of tumor-specific CTLs, the development of memory T cells, and a robust, long-lasting immune response against tumor antigens.

Cross-presentation was first scientifically described in 1976 by Michael J. Bevan, who introduced the concept of “cross-priming” (175). Bevan demonstrated that CD8^+^ T cells could be primed by antigens not synthesized within antigen-presenting cells but derived from other cells. This provided the first experimental evidence that exogenous antigens could enter the MHC class I or biosynthetic pathway, a process later termed cross-presentation. Since the 1990s, the term cross-presentation has been widely used to describe the mechanistic routing of antigens to MHC class I, while cross-priming refers specifically to the activation of CD8^+^ T cells via this pathway. By 2010, specialized dendritic cell subsets responsible for cross-presentation were characterized, and by 2020, the role of cross-presentation in cancer immunotherapy and neoantigen vaccines was clearly demonstrated.

Modern cancer vaccines are designed to enhance cross-presentation, to promote T-cell-based protective mechanisms, in addition to specific antibodies. Strategies include: Adjuvants – TLR agonists (e.g., CpG, poly I:C) and STING agonists to activate dendritic cells; delivery platforms – nanoparticles and liposomes that promote antigen uptake and routing or targeting antibodies, e.g., DEC-205, which direct antigens to DCs and favor cross-presentation pathways.

Clinical and preclinical evidence supports the importance of cross-presentation in cancer vaccination: Braun et al., demonstrated in a Phase 1 study of personalized neoantigen vaccines in resectable high-risk RCC, that the vaccine elicited robust CD8^+^ T-cell responses, expansion of peripheral T-cell clones, and reactivity against autologous tumors. Vaccine-specific T cells persisted for up to 3 years, with recognition of the patient’s own tumor confirmed in biopsy specimens following cutaneous delayed-type hypersensitivity testing. These results provide direct clinical evidence that exogenous neoantigen vaccination can prime cytotoxic T cells (170). In a melanoma preclinical model and human cells, Oliveira et al. showed that enhancing DC cross-presentation significantly increased tumor cell rejection, demonstrating the mechanistic importance of cross-presentation in anti-tumor immunity increase (176).

Genetic vaccines based on DNA and mRNA represent a rapidly advancing class of tumor antigen-specific immunotherapies. They deliver genetic material encoding tumor-associated or tumor-specific antigens directly to host cells, where the antigen is synthesized and presented via MHC I and II pathways. This induces a coordinated immune response involving CD8^+^ cytotoxic T cells, CD4^+^ helper T cells, cytokine production, and antibody generation.

The main advantage of this platform is its speed and simplicity: genetic constructs can be obtained quickly after the identification of tumor or neoantigens, without the need for large-scale production or purification of recombinant antigens. Their modular design allows for the inclusion of multiple antigens in a single vector, expanding the possibilities for immune system recognition and reducing the risk of tumor immune escape. The endogenous expression of antigens ensures MHC I presentation and priming of CD8^+^ T cells.

However, several limitations of genetic vaccines must be considered. The immunogenicity of DNA and mRNA vaccines depends critically on delivery efficiency and intracellular translation. Antigen expression levels cannot be precisely controlled, which may lead to variable immune responses. Prolonged antigen expression or secretion into circulation may result in the formation of immune complexes and type III immune pathology. Moreover, when tumor antigens are weakly immunogenic self-antigens, vaccine efficacy can be limited, and activated CD8^+^ cells may damage normal host cells expressing the same antigens, creating a potential risk of autoimmunity.

Innate sensing of nucleic acids through TLR and RIG-I pathways can also trigger systemic inflammatory responses such as fever, fatigue, or transient cytokine release. Experience from SARS-CoV-2 genetic vaccines have shown that immune-mediated adverse events may occur, including vaccine-induced thrombotic thrombocytopenia after adenoviral vector vaccines (177–179), myocarditis or pericarditis following mRNA vaccination (180–183) and occasional neurological complications such as Guillain–Barré syndrome or myelitis (183). These data emphasize the importance of controlled antigen expression, optimized delivery systems, and close monitoring in oncologic applications.

Several studies have explored combinations of tumor vaccines with other immunotherapies to enhance antitumor efficacy. One promising direction involves the integration of therapeutic vaccination with immune checkpoint inhibitors, such as anti-PD-1/PD-L1 or anti-CTLA-4 antibodies. This approach aims to combine the antigen-specific priming effect of vaccines with the immune-releasing capacity of checkpoint blockade, thereby improving both the magnitude and durability of T-cell responses (184–186). In preclinical models and early clinical studies, vaccination has been shown to increase intratumoral T-cell infiltration and upregulate checkpoint molecule expression, creating a favorable environment for synergistic action with checkpoint inhibitors (185, 187). Clinical trials in melanoma and other solid tumors, including RCC, have demonstrated that such combinations can enhance objective response rates and prolong progression-free survival compared with monotherapy, although the benefit remains heterogeneous across tumor types (188, 189).

In addition to checkpoint blockade, vaccines are evaluated together with cytokine-based immunomodulators such as IL-2, IL-15, and type I interferons to further support T-cell activation and survival. These strategies aim to optimize both the primary and effector phases of antitumor immunity (190, 192).

Although such combinations have shown promise in preclinical models, most clinical trials remained exploratory. Mechanistic insights into cytokine signaling and its integration with antigen-specific vaccination are still evolving (185, 187).

Thus, while vaccine-based combination therapy holds significant conceptual potential, its successful implementation in oncology practice requires a deeper understanding of the individual mechanisms and rigorous validation in systematic controlled clinical trials. Establishing the safety and efficacy of individual vaccination methods remains a key prerequisite for the rational development of future combination regimens.

## 7 Conclusion

Renal cell carcinoma (RCC) remains a serious global health problem, ranking among the fifteen most common malignancies, with notable geographical and gender-related variations in incidence. The prognosis for metastatic RCC is still poor, and current treatment options – including surgery, ablation techniques, radiotherapy, embolization, targeted therapy, and immunotherapy – have limited effectiveness, underscoring the urgent need for novel approaches. Molecular studies have revealed key driver mutations, such as VHL, PBRM1, BAP1, SETD2, and genes related to the mTOR pathway, which influence chromatin remodeling, angiogenesis, and cellular metabolism, though their clinical implications remain incompletely understood. Tumor-specific antigens (TSA) and tumor-associated antigens (TAA) present promising immunotherapeutic targets due to their ability to induce antigen-specific T-cell responses, yet the expression of TAAs in some normal tissues necessitates careful assessment of potential toxicity. Several antigens, including MUC1, CAIX, MAGE-A3, PRAME, B7-H3, and CD70, have shown immunogenicity in RCC. Emerging T-cell engager therapies targeting RCC antigens such as B7-H3, CD70 and respective bispecific constructs show particular promise. These modalities redirect patient T cells to tumors independently of MHC presentation, offering a complementary approach to vaccines that warrants investigation in combination regimens. A variety of *in vivo* research models, ranging from genetically engineered mice and zebrafish to PDX and humanized PDX, are crucial for preclinical evaluation but each has distinct advantages and limitations, particularly regarding the simulation of tumor–immune system interactions. Clinical trials of antigen-based vaccines and immunotherapies, such as peptide-based, vector-based, and dendritic-cell vaccines, have sometimes demonstrated immune activation and disease stabilization, yet no antigen-specific therapy for metastatic RCC has achieved FDA approval, largely due to the inability to demonstrate significant survival benefit in phase III trials. Future research priorities include refining the expression profiling of TSA/TAA in tumor versus healthy tissues, the evaluation of different tumor antigen-specific treatment strategies *in vitro*, ex vivo, *in vivo* in animal models and in pilot clinical trials followed by the investigation of combination strategies with other therapeutic modalities.
